# Editorial: Science Diplomacy and Sustainable Development: Perspectives From Latin America

**DOI:** 10.3389/frma.2021.756698

**Published:** 2021-09-08

**Authors:** Kleinsy Bonilla, Milena Serafim, Efraín Bámaca-López, Fátima Antonethe Castaneda Mena

**Affiliations:** ^1^ Department of Science and Technology Policies, Institute of Geosciences, State University of Campinas, Campinas, Brazil; ^2^ Institute for the Development of Higher Education in Guatemala, Member of the Directive Board, Instituto para el Desarrollo de la Educación Superior en Guatemala (INDESGUA), Guatemala City, Guatemala; ^3^ Facultad de Agronomía, Postdoctoral Researcher, Guatemala City, Guatemala; ^4^ Independent Researcher, Guatemala, Guatemala

**Keywords:** science diplomacy, Latin America, sustainable development, STI Policy, LATAM, multidisciplinarity, transdisciplinarity, Innovation Diplomacy

The current times present humanity with challenges that require actions beyond multidisciplinarity. They demand a transdisciplinary approach that collaborates scientific knowledge, policy, business dynamics and ancestral wisdom for solutions. In transdisciplinarity, Science Diplomacy (SD) is a specialty that fosters the engagement of diverse disciplines and stakeholders in the interface of science and foreign policy to convey a positive impact on societies and their inhabitants. This is particularly relevant in realities such as those in Latin America, where the pursuit of sustainable development for present and future generations posits a critical emphasis on respecting the planet and its resources. It is in this regard the Research Topic: Science Diplomacy and Sustainable Development: Perspectives from Latin America aims to explore relevant discussions on the need for science to be conceived internationally for sustainable national and global solutions. This is paramount because it enables actors from different countries and regions to engage in collaborative efforts towards shared objectives in seeking sustainable development, while working towards the 2030 sustainable development agenda. By bringing these issues to the fore it intended to balance studies that have been emerging from and concentrating on the European and North American focus. It is necessary to establish that the mechanisms to advance sustainable development depend on the characteristics of each territory. Therefore, for Latin American and Caribbean region, considering aspects such as communication, cultural belonging, indigenous wisdom from native populations, and gender could be beneficial in the advancement of knowledge and their effect on improving the lives of its communities.

The articles published as part of this Research Topic show the plurality of SD initiatives, the multiple challenges they face and the limits for implementation. These 14 articles can be divided into four groups. The first group deals with SD institutionalization strategies in the Latin American context. Science Diplomacy in Latin America and the Caribbean: Current Landscape, Challenges, and Future Perspectives (Soler) describes the diverse approaches, policies and practices adopted by Latin American and Caribbean countries at the national, sub-national, and regional levels. By documenting and illuminating best practices in the region, this paper further seeks to balance the perspective that has so far been largely concentrated on the regions of Europe and North America and contribute to future efforts and strategies for the development of sustainable science diplomacy mechanisms at the national, regional, North-South and South-South levels. In addition to this discussion, The Institutional Building of Science and Innovation Diplomacy in Latin America: toward a comprehensive interdisciplinary approach and analytical typology (da Silva, et al.) proposes, from a qualitative perspective and a multilevel typology, categories for the analysis of the emerging experiences within the framework of Science and Innovation Diplomacy in Latin America. Thus, providing a broad framework that can be used at various levels and sectors. In turn, Closing the Gap Between Emerging Initiatives and Integrated Strategies to Strengthen Science Diplomacy in Latin America (López-Vergès et al.) elaborates on the emerging efforts and mechanisms to bridge the gap between scientists and policymakers at the national and regional level. Based on national experiences, the paper proposes a way forward so that Latin America can leapfrog beyond disjointed training of individuals into integrated institutional strategies that can harness the tools of science diplomacy to enhance science-informed multilateral cooperation and enable more effective science-informed policymaking.

The second set of articles deals with discussions concerning policies, capacity building and good practices developed in emerging countries. The papers portray an interesting set of case studies, particularly from Central American countries. Institutional Capacity for Science Diplomacy in Central America (Jarquin-Solis and Mauduit) reveals several public management challenges stemming from the institutional disparity and complexity of the region, already marked by significant asymmetries of human development between the various countries. Highlighting and understanding such challenges may be helpful in developing meaningful strategies around science diplomacy for countries in the central America region. Already in turn, Science Diplomacy in Emerging Economies: A Phenomenological Analysis of the Colombian Case (Echeverría et al.) asserts that SD actors in Colombia are scattered, practices strongly related to traditional cooperation diplomatic activities are fragmented and call attention to the need to give a function to SD for capacity building. Therefore the authors conclude that better global intermediation and the development of new knowledge, in particular in promoting SD abilities in the scientific community would enhance the interaction between science and policy.

Focusing on the Ecuador experience, Science Diplomacy in Ecuador: Political Discourse and Practices Between 2007 and 2017 (Bonilla et al.) points out the different implications from the SD perspective in Ecuador; particularly, reflecting on the consistency between the political rhetoric and the policy implementation. Evidence presented in this article suggests that the political discourse materialized into concrete Science, Technology and Innovation (STI) policies that could partially explain positive transformations in various aspects of the STI context in Ecuador. Institutional strengthening, international mobility (inward and outward), increased scientific output, and foreign policy practices involving SD (which can be traced in the studied period 2007–2017) have all contributed towards positive transformations. Showing the process of strengthening and developing Panama’s scientific ecosystem, Science Diplomacy as an Umbrella Term for Science Advisory in Public and Foreign Relations in Small Developing Countries: The Case of Panama (Gittens et al.) presents the main public institution in the country–the National Secretariat for Science, Technology and Innovation (SENACYT)—and its role in promoting research. Furthermore, SENACYT Panamá is recognized as pioneering the importance of training young scientists in communication, relationship and leadership skills in SD. Ocean Science Diplomacy (Polejack and Fernandez Coelho) sets out how Latin America and the Caribbean seek, despite the limitations in terms of access to technology, to conduct marine scientific research in order to take advantage of the opportunities of the blue economy. Based on the United Nations Convention on the Law of the Sea, the paper seeks to discuss the transfer of marine technology to developing countries and thus advance their knowledge of this reality and promote their sustainable development. Finally, Decolonizing Science Diplomacy: A Case Study of the Dominican Republic’s COVID-19 Response (Mencía-Ripley et al.) discusses a case of how science diplomacy and a relatively new law fostering public-private partnerships allowed a university to play a major role in public health response while generating knowledge to inform public policy decisions in an unprecedented manner in the Dominican Republic. In the case, SD is discussed in the context of decolonization and the importance of the local gaze when creating academic partnerships in the context of global health emergencies.

The third set of articles seeks to debate aspects related to the education and career of researchers from Latin American and Caribbean countries, with special emphasis on the issue of gender, analyzing the participation of women in the scientific community. Science diplomacy for climate action and sustainable development in Latin America and the Caribbean: How important is the Early Career perspective to New Governance? (Cuellar-Ramirez) collects a series of examples of the progress, best practices, gaps, challenges and solutions. The author does it from the perspective of Early Careers Researchers (ECRs) and undergraduate and graduate students, highlighting what it has been done to engage scientists in society-policy-science interaction for sustainable development agenda and climate action in Latin America and the Caribbean. The case of Call to Action: Supporting Latin American Early Career Researchers on the Quest for Sustainable Development in the Region (Lopez-Verges et al.) encourages reflection on the challenges that the ECRs in Latin America and Caribbeans Countries experience. By considering these challenges and actively participating in studies about ECRs, she proposes the creation of strategies to better support the next generation of science change-makers in the region. The success of this study required collaboration between ECR organizations and policymakers, and therefore harnessing the human capital that ECRs-LAC represents is crucial for the region to meet the United Nations (UN) 2030 sustainable development goals. Looking for the gender perspective, Participation in Communities of Women Scientists in Central America: Implications From the Science Diplomacy Perspective (Bonilla et al.) explores the experiences of women scientists participating in communities of Guatemala, El Salvador, Honduras and Panama, and attempt to systematize the challenges and opportunities derived from such activities. The findings of this study revealed few cases of community building experiences among women scientists within the studied countries. Evidence also showed the emergence of shared patterns in terms of barriers and disincentives to participating in such communities. Therefore, offering explanation for the lack of participation of women scientists in collective initiatives in Central America.

Finally, the fourth set of papers deals with discussions related to the communication of science, from the perspective of building bridges with the DS debates. The first one, Bringing Policymakers to Science Through Communication: A Perspective From Latin America (Pulido-Salgado and Castaneda Mena), aims at providing some recommendations to build bridges between science and decision-making parties through communication, and by exploring how Latin American diplomats and policymakers engage with scientific knowledge. In turn, Latin American Network for Scientific Culture (RedLCC): A Regional Science Communication Initiative (Moronta-Barrios et al.) presents an analysis of blogs and social media platforms, that are specifically open for addressing information about science to citizens in the Latin America, and are easily accessible resources. In addition, the Latin American Network for Scientific Culture (RedLCC) is highlighted as one of the regional science communication initiatives. It brings together regional scientists that communicate science for Latin American communities, and consequently nurtures the “Science for Diplomacy” dimension of SD. Thus, the communication mechanisms for Science Diplomacy and Sustainable Development are essential and strengthening them progressively could contribute to effective science communication.

The co-editors are convinced that these articles can contribute to future efforts and strategies for the development of sustainable science diplomacy mechanisms at the national, regional, North-South and South-South levels. We believe that a major contribution of the Research Topic is the inclusion of the different approaches, policies and practices adopted by Latin American and Caribbean countries at different levels. In addition, this Research Topic contributes to increasing the visibility of SD studies in the scope of Latin America, allowing for counterbalancing the emphasis, focus and narratives that studies from the regions of Europe and America imprint on DS studies. We look forward to seeing the growth of the field of Latin American studies in science diplomacy.

We thank all the authors, co-authors, reviewers and external editors who participated in this process. We also thank Frontiers in Research Metrics and Analytics for making it possible the publication of this Research Topic and all the articles as part of this collection.

